# Institutional Outdoor Work and Administration

**Published:** 1911-11-25

**Authors:** 


### INSTITUTIONAL OUTDOOR WORK AND ADMINISTRATION.

### VI.—Conclusion.

Readers having borne with the foregoing olio of practical hints regarding the development of the estate of a small medical institution, one is emboldened to make a few general observations in conclusion. The plan does not presuppose a resident administrator. If a house committee member of activity and large leisure live in the vicinity he may obtain much daily interesting occupation in this way, with considerable benefit to his institution. But the work must be a "one man show," whoever sees to it. And perhaps—a suggestion rather unpopular in these days—it is badly suited to the feminine genius. Some of the details of administration described may have seemed to border upon the niggling, but there is in all probability a period of temporary financial closeness ahead of all charities. Initiated in the past by rich men, often, as is so commonly the case, under clerical influence, and planned rather too much, perhaps, in accordance with the private emotional dictate of pity for the suffering, and with too much regard for appearance, they have now to come into line with a clearer perception of the needs of the community and an upheaval of economic ideas. That impersonal claim of the municipality for strict economy which is strongly visible in our asylums may cause flower beds to go out of fashion elsewhere. The obligation holds good, at any rate, in the case of institutions designed to deal with the great popular disease, tuberculosis. Here the idea of making funds go as far as possible must override at once every other consideration. For instance, a certain otherwise exemplarily administered institution of this nature is about to build a chapel, being already in possession of common-room accommodation for its inmates. The splendid organising powers of its superintendent might surely be better employed.

### Rural Repopulation.

The above is another great principle the ægis of which may be invoked by little enterprises whose furtherance has been the object of the present endeavour. Increasing urbanisation, a necessary evil of modern civilisation, may be minimised in one direction very rationally by taking weaklings from the city and planting them upon the land. We are not ready yet for such an ambitious scheme (noticed in The Hospital, January 14, 1911) as complete rural communities of the tuberculous, under a new hierarchy of doctors; but any practical means of remedying the common case of a working-class convalescent consumptive returning to the factory to break down again, or to loafing and starving while employers fight shy of him, is too important to be neglected at a time when, although we hear regularly every few months of a certain specific remedy, it is unfortunately always a fresh one. The re-establishment of general health and the powers of the constitution, important in the treatment of any diseased condition, is so peculiarly so in that of tuberculosis that for many years yet it must remain the very first therapeutic indication.

### Possibilities of Growth.

When the matter is thus viewed, then, and while, in spite of talk about the claims of preventive work, the individual consumptive remains a problem not to be dodged, it seems reasonable that farming developments in some degree should be made conditional to the establishment or extension of sanatoriums under the proposed National Insurance Bill. Each institution would push its operations in directions indicated by individual opportunities and success, while an interchange of experience would soon define the best available general plan. Mr. Rider Haggard's account of the Salvation Army Colony at Hadleigh probably foreshadows something like a further result, with each department of agriculture under supervision of a lay expert. But the foundations must be laid and the beginnings of the work tackled, in our present case, by those whose eyes are likely to have met with the disjointed remarks now happily concluded.

